# Redetermined structure of *N*,*N*-dibutyl-4-[(*E*)-2-(4-{(*E*)-2-[4-(di­butyl­amino)­phen­yl]ethen­yl}-2,5-di­meth­oxy­phen­yl)ethen­yl]aniline

**DOI:** 10.1107/S2414314626011053

**Published:** 2026-10-30

**Authors:** Dieter Schollmeyer, Heiner Detert

**Affiliations:** a University of Mainz, Department of Chemistry, Duesbergweg 10-14, 55099 Mainz, Germany; University of Aberdeen, United Kingdom

**Keywords:** crystal structure, distyryl­benzene, stilbene, phenyl­ene vinyl­ene

## Abstract

The title compound, C_40_H_56_N_2_O_2_, was prepared *via* a Horner olefination reaction. Contrary to the previous structure, in which the complete mol­ecule was generated by inversion symmetry and displayed unreasonable geometrical features [Zhang *et al.* (2005 ▸). *Chin. J. Struct. Chem.***24**, 669–672], the mol­ecule has *C*_1_ symmetry and the dihedral angles between the central and peripheral aromatic rings are 39.29 (9) and 18.95 (9)°. A weak C—H⋯π inter­action occurs in the extended structure.

## Abstract 2

The title compound was prepared *via* a Horner olefination reaction. Contrary to the previous structure, in which the complete mol­ecule was generated by inversion symmetry and displayed unreasonable geometrical features, the mol­ecule has *C*_1_ symmetry and the dihedral angles between the central and peripheral aromatic rings are 39.29 (9) and 18.95 (9)°. A weak C—H⋯π inter­action occurs in the extended structure.

## Graphical abstract

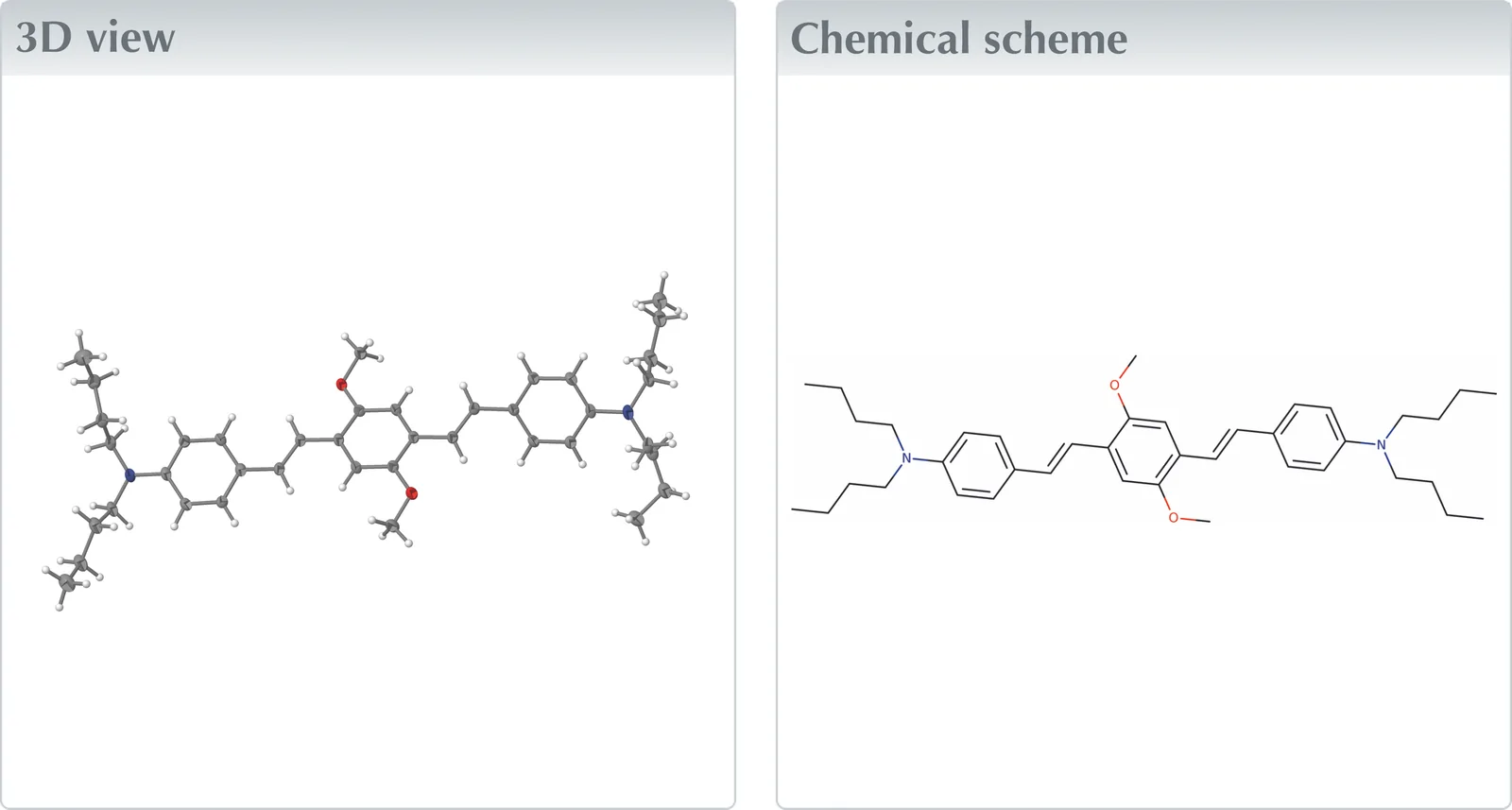

## Structure description

The title compound, C_40_H_56_N_2_O_2_ (**I**), is a linear *para*-distyryl­benzene, which was prepared in a project on phenyl­ene­vinyl­ene dyes (Stalmach & Detert, 2000 ▸) with focus on increased two-photon absorption (Detert *et al.*, 2004 ▸, 2006 ▸; Strehmel *et al.*, 2003 ▸) and acidochromic fluoro­phors (Moschel *et al.*, 2026 ▸; Detert & Schmitt, 2004 ▸; Schmitt *et al.*, 2013 ▸). The fundamental chromophore, 1,4-bis-(4-di­butyl­amino­styr­yl)benzene had been investigated by Bunz (Zucchero *et al.*, 2009 ▸) and a crystal structure analysis of the title compound has already been published (Zhang *et al.*, 2005 ▸; Cambridge Structural Database refcocde KARSOM). These authors obtained diffraction data at 293 K and their results are significantly different from ours, obtained at 120 K. In both cases the crystal structures belong to the triclinic space group *P*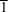 but Zhang and co-workers established a unit cell [*a* = 9.303 (2) Å, *b* = 9.625 (2) Å, *c* = 10.442 (2) Å, *α* = 81.493 (12)°, *β* = 78.387 (12)°, *γ* = 87.876 (11)°, *V* = 905.75 Å^3^] with approximately half the volume of (**I**) (Table 2 ▸ ▸) and therefore a complete mol­ecule generated by crystallographic inversion symmetry. The alkyl chains in KARSOM show high anisotropic displacement parameters (ADPs) and C—C bond lengths far from their expected values.

Our investigation reveals a mol­ecule without symmetry (Fig. 1 ▸), normal ADPs and standard bond lengths in the alkyl chains. The mol­ecule is composed of five almost planar units, the dihedral angles between the C1–C6 central ring and ring 2 (C9–C14) is 39.29 (9)° and between the central ring and ring 3 (C17–C22) is 18.95 (9)°. The dihedral angle between the planes of rings 2 and 3 amounts to 20.65 (9)°. The torsion angles between the central ring and adjacent vinyl­ene groups C3—C2—C7—C8 of 21.2 (3)° and C10—C9—C8—C7 of 17.1 (3)° indicate significant conjugation along the π-system. The angle sum at N27 of 358.18 (17)° and of 359.88 (17)° at N36 and the short bond lengths of C12—N27 [1.384 (2) Å] and C20—N36 [1.386 (2) Å] result from strong inter­action between the nitro­gen atoms and adjacent phenyl ring. Whereas the oxygen atoms of the meth­oxy groups are almost in the plane of ring 1, the methyl carbon atom C24 is −0.132 (3) Å below, whereas atom C26 is 0.211 (3) Å above that plane. The unit cell contains two mol­ecules, separated by the terminal alkyl chains. While three alkyl chains are *all-trans*, the fourth has a *gauche* conformation with a torsion angle C28—C29—C30—C31 of 72.4 (2)°. In the extended structure, one weak C—H⋯π inter­action (Table 1 ▸) occurs. The packing of (**I**) is shown in Fig. 2 ▸.

## Synthesis and crystallization

The title compound was obtained in a Horner olefination of 1,4-bis­(di­eth­oxy­phospho­rylmeth­yl)-2,5-di­meth­oxy­benzene (Brehm *et al.*, 2002 ▸) and 4-di­butyl­amino­benzaldehyde in tetra­hydro­furan using KOtBu as a base in 67% yield. Recrystallization from a solvent mixture of 2-propanol and chloro­form by slow evaporation yielded orange needles of (**I**) with m.p. = 407 K.

## Refinement

Crystal data, data collection and structure refinement details are summarized in Table 2 ▸.

## Supplementary Material

Crystal structure: contains datablock(s) I, global. DOI: 10.1107/S2414314626011053/hb4579sup1.cif

Structure factors: contains datablock(s) I. DOI: 10.1107/S2414314626011053/hb4579Isup2.hkl

Supporting information file. DOI: 10.1107/S2414314626011053/hb4579Isup3.cml

CCDC reference: 2597963

Additional supporting information:  crystallographic information; 3D view; checkCIF report

## Figures and Tables

**Figure 1:**
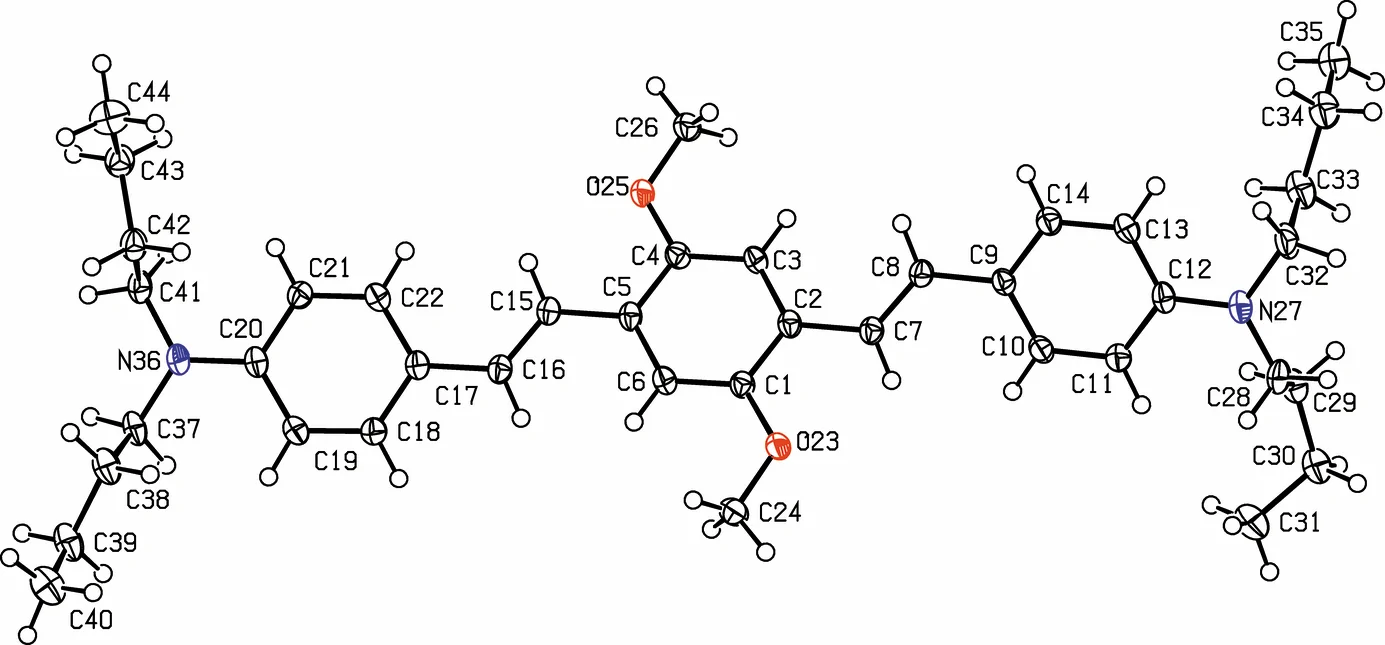
The mol­ecular structure of (**I**) with displacement ellipsoids drawn at the 50% probability level.

**Figure 2:**
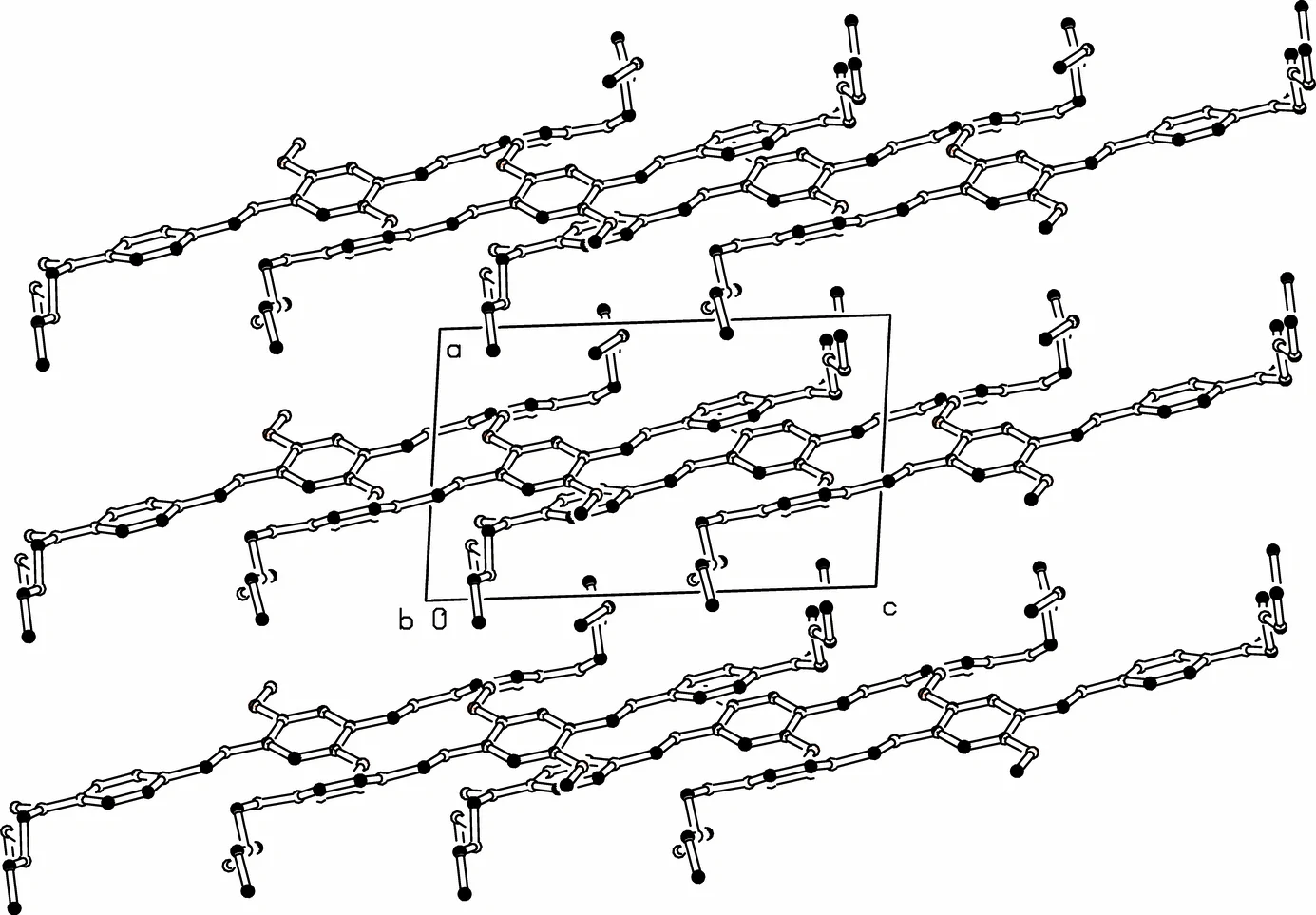
The packing for (**I**) viewed along *b*-axis direction with hydrogen atoms removed for clarity.

**Table 1:** Hydrogen-bond geometry (Å, °) *Cg*3 is the centroid of the C17–C22 ring.

*D*—H⋯*A*	*D*—H	H⋯*A*	*D*⋯*A*	*D*—H⋯*A*
C30—H30*B*⋯*Cg*3^i^	0.99	2.78	3.719 (2)	158

Symmetry code: (i) 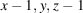.

**Table 2:** Experimental details

Crystal data	
Chemical formula	C_40_H_56_N_2_O_2_
*M*_r_	596.86
Crystal system, space group	Triclinic, *P* 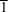
Temperature (K)	120
*a*, *b*, *c* (Å)	9.2000 (3), 12.5077 (5), 15.1438 (6)
α, β, γ (°)	88.387 (3), 85.054 (3), 83.850 (3)
*V* (Å^3^)	1725.82 (11)
*Z*	2
Radiation type	Mo *K*α
μ (mm^−1^)	0.07
Crystal size (mm)	0.88 × 0.16 × 0.13

Data collection	
Diffractometer	Stoe IPDS 2T
No. of measured, independent and observed [*I* > 2σ(*I*)] reflections	14720, 8089, 6190
*R*_int_	0.054
(sin θ/λ)_max_ (Å^−1^)	0.659

Refinement	
*R*[*F*^2^ > 2σ(*F*^2^)], *wR*(*F*^2^), *S*	0.069, 0.193, 1.06
No. of reflections	8089
No. of parameters	403
H-atom treatment	H-atom parameters constrained
Δρ_max_, Δρ_min_ (e Å^−3^)	0.59, −0.27

Computer programs: *X-AREAWinXpose*, *X-AREARecipe* and *X-AREAIntegrate* (Stoe, 2020 ▸), *SHELXT2014* (Sheldrick, 2015*a* ▸), *SHELXL2019/2* (Sheldrick, 2015*b* ▸) and *PLATON* (Spek, 2009 ▸).

## full crystallographic data

### N,N-Dibutyl-4-[(E)-2-(4-{(E)-2-[4-(dibutylamino)phenyl]ethenyl}-2,5-dimethoxyphenyl)ethenyl]aniline Crystal data

**Table:**

C_40_H_56_N_2_O_2_	*F*(000) = 652
*M**_r_* = 596.86	*D*_x_ = 1.149 Mg m^−^^3^
Triclinic, *P*1	Melting point: 403 K
*a* = 9.2000 (3) Å	Mo *K*α radiation, λ = 0.71073 Å
*b* = 12.5077 (5) Å	Cell parameters from 15472 reflections
*c* = 15.1438 (6) Å	θ = 2.9–28.3°
α = 88.387 (3)°	µ = 0.07 mm^−^^1^
β = 85.054 (3)°	*T* = 120 K
γ = 83.850 (3)°	Needle, orange
*V* = 1725.82 (11) Å^3^	0.88 × 0.16 × 0.13 mm
*Z* = 2	

### N,N-Dibutyl-4-[(E)-2-(4-{(E)-2-[4-(dibutylamino)phenyl]ethenyl}-2,5-dimethoxyphenyl)ethenyl]aniline Data collection

**Table:**

Stoe IPDS 2T diffractometer	*R*_int_ = 0.054
Detector resolution: 6.67 pixels mm^-1^	θ_max_ = 28.0°, θ_min_ = 2.9°
rotation method, ω scans	*h* = −12→12
14720 measured reflections	*k* = −16→16
8089 independent reflections	*l* = −19→19
6190 reflections with *I* > 2σ(*I*)	

### N,N-Dibutyl-4-[(E)-2-(4-{(E)-2-[4-(dibutylamino)phenyl]ethenyl}-2,5-dimethoxyphenyl)ethenyl]aniline Refinement

**Table:**

Refinement on *F*^2^	Primary atom site location: dual
Least-squares matrix: full	Hydrogen site location: inferred from neighbouring sites
*R*[*F*^2^ > 2σ(*F*^2^)] = 0.069	H-atom parameters constrained
*wR*(*F*^2^) = 0.193	*w* = 1/[σ^2^(*F*_o_^2^) + (0.0797*P*)^2^ + 1.3801*P*] where *P* = (*F*_o_^2^ + 2*F*_c_^2^)/3
*S* = 1.06	(Δ/σ)_max_ < 0.001
8089 reflections	Δρ_max_ = 0.59 e Å^−^^3^
403 parameters	Δρ_min_ = −0.27 e Å^−^^3^
0 restraints	

### N,N-Dibutyl-4-[(E)-2-(4-{(E)-2-[4-(dibutylamino)phenyl]ethenyl}-2,5-dimethoxyphenyl)ethenyl]aniline Special details

**Table:**

Geometry. All esds (except the esd in the dihedral angle between two l.s. planes) are estimated using the full covariance matrix. The cell esds are taken into account individually in the estimation of esds in distances, angles and torsion angles; correlations between esds in cell parameters are only used when they are defined by crystal symmetry. An approximate (isotropic) treatment of cell esds is used for estimating esds involving l.s. planes.
Refinement. The hydrogen atoms were placed at calculated positions and were refined in the riding-model approximation with C—H = 0.95–0.99 Å, and with *U*_iso_(H) = 1.2 *U*_eq_(C).

### N,N-Dibutyl-4-[(E)-2-(4-{(E)-2-[4-(dibutylamino)phenyl]ethenyl}-2,5-dimethoxyphenyl)ethenyl]aniline Fractional atomic coordinates and isotropic or equivalent isotropic displacement parameters (Å^2^)

**Table:**

	*x*	*y*	*z*	*U*_iso_*/*U*_eq_	
C1	0.5408 (2)	0.17863 (15)	0.18429 (12)	0.0211 (4)	
C2	0.4564 (2)	0.27001 (15)	0.15316 (12)	0.0206 (4)	
C3	0.3984 (2)	0.34755 (15)	0.21495 (12)	0.0211 (4)	
H3	0.339671	0.409521	0.195679	0.025*	
C4	0.4246 (2)	0.33605 (15)	0.30392 (12)	0.0210 (4)	
C5	0.5087 (2)	0.24413 (15)	0.33530 (12)	0.0205 (4)	
C6	0.5667 (2)	0.16688 (15)	0.27307 (12)	0.0222 (4)	
H6	0.625367	0.104783	0.292184	0.027*	
C7	0.4282 (2)	0.27920 (15)	0.05938 (12)	0.0214 (4)	
H7	0.441200	0.214621	0.026749	0.026*	
C8	0.3856 (2)	0.37008 (15)	0.01510 (12)	0.0212 (4)	
H8	0.377928	0.435596	0.046238	0.025*	
C9	0.3500 (2)	0.37615 (15)	−0.07761 (12)	0.0203 (4)	
C10	0.3199 (2)	0.28679 (15)	−0.12356 (13)	0.0233 (4)	
H10	0.319156	0.219503	−0.092914	0.028*	
C11	0.2911 (2)	0.29226 (16)	−0.21145 (13)	0.0249 (4)	
H11	0.270430	0.229335	−0.239610	0.030*	
C12	0.2920 (2)	0.39028 (16)	−0.26036 (12)	0.0232 (4)	
C13	0.3159 (2)	0.48127 (16)	−0.21361 (13)	0.0242 (4)	
H13	0.312682	0.549314	−0.243273	0.029*	
C14	0.3443 (2)	0.47392 (15)	−0.12493 (13)	0.0231 (4)	
H14	0.360273	0.537217	−0.095541	0.028*	
C15	0.5355 (2)	0.23301 (15)	0.42904 (12)	0.0213 (4)	
H15	0.513547	0.295307	0.464194	0.026*	
C16	0.5886 (2)	0.14192 (16)	0.46962 (13)	0.0229 (4)	
H16	0.605244	0.079182	0.434704	0.027*	
C17	0.6235 (2)	0.12953 (15)	0.56214 (12)	0.0218 (4)	
C18	0.6739 (2)	0.02830 (16)	0.59628 (13)	0.0241 (4)	
H18	0.684697	−0.031714	0.558302	0.029*	
C19	0.7085 (2)	0.01233 (16)	0.68303 (13)	0.0249 (4)	
H19	0.742791	−0.057753	0.703073	0.030*	
C20	0.6936 (2)	0.09848 (16)	0.74206 (13)	0.0230 (4)	
C21	0.6453 (2)	0.20119 (16)	0.70739 (13)	0.0239 (4)	
H21	0.636397	0.261794	0.744651	0.029*	
C22	0.6109 (2)	0.21514 (15)	0.62092 (13)	0.0237 (4)	
H22	0.577532	0.285201	0.600378	0.028*	
O23	0.59712 (16)	0.10492 (11)	0.12092 (9)	0.0260 (3)	
C24	0.6693 (3)	0.00613 (16)	0.15097 (14)	0.0315 (5)	
H24A	0.701147	−0.039992	0.100168	0.047*	
H24B	0.601560	−0.030108	0.192167	0.047*	
H24C	0.754925	0.020349	0.181301	0.047*	
O25	0.36914 (16)	0.41065 (11)	0.36682 (9)	0.0254 (3)	
C26	0.3031 (3)	0.51089 (16)	0.33501 (14)	0.0294 (4)	
H26A	0.373547	0.543800	0.293306	0.044*	
H26B	0.273955	0.558826	0.384991	0.044*	
H26C	0.216302	0.499047	0.304832	0.044*	
N27	0.27219 (19)	0.39505 (14)	−0.35003 (11)	0.0267 (4)	
C28	0.2330 (2)	0.30169 (17)	−0.39484 (13)	0.0269 (4)	
H28A	0.296849	0.237558	−0.376505	0.032*	
H28B	0.254255	0.312366	−0.459504	0.032*	
C29	0.0729 (2)	0.27857 (17)	−0.37704 (14)	0.0287 (4)	
H29A	0.044012	0.284471	−0.312721	0.034*	
H29B	0.009833	0.333786	−0.408235	0.034*	
C30	0.0460 (2)	0.16768 (18)	−0.40697 (15)	0.0322 (5)	
H30A	0.090515	0.157162	−0.468397	0.039*	
H30B	−0.060987	0.164635	−0.407535	0.039*	
C31	0.1084 (3)	0.07616 (19)	−0.34828 (17)	0.0402 (6)	
H31A	0.084309	0.007520	−0.369697	0.060*	
H31B	0.065876	0.086341	−0.287132	0.060*	
H31C	0.215233	0.075738	−0.350254	0.060*	
C32	0.2532 (3)	0.49942 (18)	−0.39501 (14)	0.0335 (5)	
H32A	0.265660	0.488803	−0.459870	0.040*	
H32B	0.330793	0.542959	−0.379495	0.040*	
C33	0.1009 (3)	0.56239 (19)	−0.37052 (16)	0.0359 (5)	
H33A	0.026924	0.533277	−0.404250	0.043*	
H33B	0.072955	0.550797	−0.306645	0.043*	
C34	0.0981 (3)	0.68211 (19)	−0.38979 (16)	0.0365 (5)	
H34A	0.174314	0.711186	−0.357874	0.044*	
H34B	0.121511	0.694320	−0.454083	0.044*	
C35	−0.0498 (3)	0.7412 (2)	−0.36179 (16)	0.0396 (5)	
H35A	−0.046243	0.818636	−0.372208	0.059*	
H35B	−0.074823	0.727222	−0.298603	0.059*	
H35C	−0.124421	0.716231	−0.396380	0.059*	
N36	0.7233 (2)	0.08456 (14)	0.83005 (11)	0.0274 (4)	
C37	0.7528 (2)	−0.02264 (17)	0.86865 (14)	0.0280 (4)	
H37A	0.694998	−0.071906	0.839741	0.034*	
H37B	0.717213	−0.020358	0.932223	0.034*	
C38	0.9124 (2)	−0.06967 (18)	0.86115 (15)	0.0318 (5)	
H38A	0.969653	−0.025745	0.895748	0.038*	
H38B	0.951986	−0.066498	0.798351	0.038*	
C39	0.9307 (3)	−0.18629 (19)	0.89503 (16)	0.0351 (5)	
H39A	0.901197	−0.188024	0.959441	0.042*	
H39B	0.863940	−0.227971	0.865385	0.042*	
C40	1.0861 (3)	−0.2400 (2)	0.87906 (19)	0.0431 (6)	
H40A	1.118263	−0.235081	0.815815	0.065*	
H40B	1.089034	−0.315771	0.897763	0.065*	
H40C	1.151472	−0.203793	0.913283	0.065*	
C41	0.7156 (2)	0.17782 (17)	0.88650 (13)	0.0272 (4)	
H41A	0.712800	0.152539	0.949102	0.033*	
H41B	0.622510	0.223243	0.878719	0.033*	
C42	0.8420 (2)	0.24723 (17)	0.86880 (14)	0.0277 (4)	
H42A	0.928722	0.212788	0.897095	0.033*	
H42B	0.868577	0.251771	0.804146	0.033*	
C43	0.8026 (2)	0.36011 (18)	0.90428 (15)	0.0310 (5)	
H43A	0.771205	0.354869	0.968303	0.037*	
H43B	0.718364	0.395131	0.874008	0.037*	
C44	0.9281 (3)	0.4306 (2)	0.89166 (19)	0.0413 (6)	
H44A	0.959686	0.436647	0.828406	0.062*	
H44B	1.010474	0.398327	0.923725	0.062*	
H44C	0.894861	0.502346	0.914733	0.062*	

### N,N-Dibutyl-4-[(E)-2-(4-{(E)-2-[4-(dibutylamino)phenyl]ethenyl}-2,5-dimethoxyphenyl)ethenyl]aniline Atomic displacement parameters (Å^2^)

**Table:**

	*U*^11^	*U*^22^	*U*^33^	*U*^12^	*U*^13^	*U*^23^
C1	0.0228 (9)	0.0225 (9)	0.0177 (9)	−0.0026 (7)	0.0000 (7)	0.0014 (7)
C2	0.0212 (9)	0.0233 (9)	0.0177 (9)	−0.0047 (7)	−0.0022 (7)	0.0026 (7)
C3	0.0224 (9)	0.0222 (9)	0.0181 (9)	−0.0016 (7)	−0.0013 (7)	0.0045 (7)
C4	0.0222 (9)	0.0217 (9)	0.0187 (9)	−0.0034 (7)	0.0009 (7)	0.0027 (7)
C5	0.0203 (8)	0.0254 (9)	0.0164 (9)	−0.0054 (7)	−0.0021 (7)	0.0026 (7)
C6	0.0230 (9)	0.0244 (9)	0.0187 (9)	−0.0010 (7)	−0.0022 (7)	0.0037 (7)
C7	0.0233 (9)	0.0239 (9)	0.0172 (9)	−0.0039 (7)	−0.0015 (7)	0.0010 (7)
C8	0.0222 (9)	0.0242 (9)	0.0171 (9)	−0.0021 (7)	−0.0008 (7)	−0.0006 (7)
C9	0.0210 (8)	0.0221 (9)	0.0168 (9)	0.0006 (7)	−0.0007 (7)	0.0023 (7)
C10	0.0264 (9)	0.0225 (9)	0.0204 (9)	0.0005 (7)	−0.0031 (7)	0.0037 (7)
C11	0.0288 (10)	0.0248 (9)	0.0208 (9)	−0.0004 (8)	−0.0032 (8)	0.0001 (7)
C12	0.0214 (9)	0.0307 (10)	0.0166 (9)	−0.0002 (7)	−0.0013 (7)	0.0035 (7)
C13	0.0257 (9)	0.0250 (9)	0.0216 (9)	−0.0034 (7)	−0.0019 (7)	0.0065 (7)
C14	0.0245 (9)	0.0239 (9)	0.0214 (9)	−0.0037 (7)	−0.0033 (7)	0.0026 (7)
C15	0.0224 (9)	0.0249 (9)	0.0167 (9)	−0.0030 (7)	−0.0014 (7)	0.0000 (7)
C16	0.0249 (9)	0.0263 (9)	0.0179 (9)	−0.0045 (7)	−0.0023 (7)	0.0003 (7)
C17	0.0213 (9)	0.0259 (9)	0.0181 (9)	−0.0027 (7)	−0.0018 (7)	0.0018 (7)
C18	0.0283 (10)	0.0233 (9)	0.0209 (9)	−0.0032 (7)	−0.0027 (7)	0.0002 (7)
C19	0.0273 (10)	0.0242 (9)	0.0229 (10)	−0.0018 (7)	−0.0041 (8)	0.0051 (7)
C20	0.0222 (9)	0.0288 (10)	0.0181 (9)	−0.0034 (7)	−0.0025 (7)	0.0043 (7)
C21	0.0277 (10)	0.0247 (9)	0.0187 (9)	0.0004 (7)	−0.0016 (7)	−0.0020 (7)
C22	0.0254 (9)	0.0238 (9)	0.0218 (9)	−0.0011 (7)	−0.0035 (7)	0.0039 (7)
O23	0.0339 (8)	0.0235 (7)	0.0191 (7)	0.0039 (6)	−0.0029 (6)	0.0005 (5)
C24	0.0426 (12)	0.0246 (10)	0.0244 (10)	0.0062 (9)	−0.0001 (9)	0.0015 (8)
O25	0.0354 (8)	0.0240 (7)	0.0160 (7)	0.0014 (6)	−0.0025 (6)	0.0013 (5)
C26	0.0403 (12)	0.0253 (10)	0.0206 (10)	0.0036 (8)	−0.0006 (8)	0.0006 (7)
N27	0.0307 (9)	0.0323 (9)	0.0174 (8)	−0.0038 (7)	−0.0043 (7)	0.0044 (7)
C28	0.0271 (10)	0.0385 (11)	0.0148 (9)	−0.0030 (8)	−0.0007 (7)	0.0001 (8)
C29	0.0250 (10)	0.0363 (11)	0.0236 (10)	−0.0003 (8)	−0.0012 (8)	0.0045 (8)
C30	0.0307 (11)	0.0373 (12)	0.0286 (11)	−0.0050 (9)	−0.0041 (9)	0.0070 (9)
C31	0.0440 (13)	0.0361 (12)	0.0401 (13)	−0.0030 (10)	−0.0054 (11)	0.0104 (10)
C32	0.0431 (13)	0.0381 (12)	0.0201 (10)	−0.0067 (10)	−0.0070 (9)	0.0100 (8)
C33	0.0340 (11)	0.0421 (12)	0.0323 (12)	−0.0082 (9)	−0.0068 (9)	0.0116 (10)
C34	0.0377 (12)	0.0416 (12)	0.0311 (12)	−0.0077 (10)	−0.0058 (9)	0.0057 (9)
C35	0.0432 (13)	0.0437 (13)	0.0320 (12)	−0.0046 (10)	−0.0047 (10)	0.0038 (10)
N36	0.0371 (10)	0.0283 (9)	0.0169 (8)	−0.0037 (7)	−0.0047 (7)	0.0047 (6)
C37	0.0304 (10)	0.0336 (11)	0.0202 (9)	−0.0049 (8)	−0.0045 (8)	0.0103 (8)
C38	0.0287 (10)	0.0389 (12)	0.0278 (11)	−0.0055 (9)	−0.0026 (8)	0.0104 (9)
C39	0.0337 (11)	0.0410 (12)	0.0308 (11)	−0.0079 (9)	−0.0045 (9)	0.0138 (9)
C40	0.0388 (13)	0.0444 (14)	0.0453 (15)	−0.0005 (11)	−0.0077 (11)	0.0099 (11)
C41	0.0292 (10)	0.0357 (11)	0.0162 (9)	−0.0016 (8)	−0.0014 (7)	0.0020 (8)
C42	0.0250 (10)	0.0352 (11)	0.0219 (10)	0.0013 (8)	−0.0015 (8)	−0.0009 (8)
C43	0.0306 (11)	0.0351 (11)	0.0263 (11)	0.0004 (9)	−0.0016 (8)	−0.0016 (8)
C44	0.0334 (12)	0.0398 (13)	0.0505 (15)	−0.0047 (10)	−0.0001 (11)	−0.0066 (11)

### N,N-Dibutyl-4-[(E)-2-(4-{(E)-2-[4-(dibutylamino)phenyl]ethenyl}-2,5-dimethoxyphenyl)ethenyl]aniline Geometric parameters (Å, º)

**Table:**

C1—O23	1.379 (2)	N27—C28	1.460 (3)
C1—C6	1.387 (3)	C28—C29	1.531 (3)
C1—C2	1.405 (3)	C28—H28A	0.9900
C2—C3	1.398 (3)	C28—H28B	0.9900
C2—C7	1.465 (3)	C29—C30	1.523 (3)
C3—C4	1.390 (3)	C29—H29A	0.9900
C3—H3	0.9500	C29—H29B	0.9900
C4—O25	1.379 (2)	C30—C31	1.523 (3)
C4—C5	1.410 (3)	C30—H30A	0.9900
C5—C6	1.400 (3)	C30—H30B	0.9900
C5—C15	1.462 (3)	C31—H31A	0.9800
C6—H6	0.9500	C31—H31B	0.9800
C7—C8	1.342 (3)	C31—H31C	0.9800
C7—H7	0.9500	C32—C33	1.553 (3)
C8—C9	1.467 (3)	C32—H32A	0.9900
C8—H8	0.9500	C32—H32B	0.9900
C9—C10	1.398 (3)	C33—C34	1.515 (3)
C9—C14	1.398 (3)	C33—H33A	0.9900
C10—C11	1.378 (3)	C33—H33B	0.9900
C10—H10	0.9500	C34—C35	1.511 (3)
C11—C12	1.415 (3)	C34—H34A	0.9900
C11—H11	0.9500	C34—H34B	0.9900
C12—N27	1.384 (2)	C35—H35A	0.9800
C12—C13	1.404 (3)	C35—H35B	0.9800
C13—C14	1.389 (3)	C35—H35C	0.9800
C13—H13	0.9500	N36—C37	1.456 (2)
C14—H14	0.9500	N36—C41	1.458 (3)
C15—C16	1.344 (3)	C37—C38	1.518 (3)
C15—H15	0.9500	C37—H37A	0.9900
C16—C17	1.464 (3)	C37—H37B	0.9900
C16—H16	0.9500	C38—C39	1.529 (3)
C17—C22	1.401 (3)	C38—H38A	0.9900
C17—C18	1.402 (3)	C38—H38B	0.9900
C18—C19	1.382 (3)	C39—C40	1.516 (3)
C18—H18	0.9500	C39—H39A	0.9900
C19—C20	1.407 (3)	C39—H39B	0.9900
C19—H19	0.9500	C40—H40A	0.9800
C20—N36	1.386 (2)	C40—H40B	0.9800
C20—C21	1.415 (3)	C40—H40C	0.9800
C21—C22	1.375 (3)	C41—C42	1.525 (3)
C21—H21	0.9500	C41—H41A	0.9900
C22—H22	0.9500	C41—H41B	0.9900
O23—C24	1.421 (2)	C42—C43	1.521 (3)
C24—H24A	0.9800	C42—H42A	0.9900
C24—H24B	0.9800	C42—H42B	0.9900
C24—H24C	0.9800	C43—C44	1.522 (3)
O25—C26	1.423 (2)	C43—H43A	0.9900
C26—H26A	0.9800	C43—H43B	0.9900
C26—H26B	0.9800	C44—H44A	0.9800
C26—H26C	0.9800	C44—H44B	0.9800
N27—C32	1.456 (3)	C44—H44C	0.9800
			
O23—C1—C6	123.45 (16)	C28—C29—H29A	109.0
O23—C1—C2	115.66 (16)	C30—C29—H29B	109.0
C6—C1—C2	120.87 (17)	C28—C29—H29B	109.0
C3—C2—C1	117.67 (17)	H29A—C29—H29B	107.8
C3—C2—C7	122.60 (17)	C29—C30—C31	113.54 (19)
C1—C2—C7	119.70 (17)	C29—C30—H30A	108.9
C4—C3—C2	121.52 (17)	C31—C30—H30A	108.9
C4—C3—H3	119.2	C29—C30—H30B	108.9
C2—C3—H3	119.2	C31—C30—H30B	108.9
O25—C4—C3	123.21 (16)	H30A—C30—H30B	107.7
O25—C4—C5	115.93 (16)	C30—C31—H31A	109.5
C3—C4—C5	120.85 (17)	C30—C31—H31B	109.5
C6—C5—C4	117.34 (17)	H31A—C31—H31B	109.5
C6—C5—C15	122.35 (17)	C30—C31—H31C	109.5
C4—C5—C15	120.30 (17)	H31A—C31—H31C	109.5
C1—C6—C5	121.74 (17)	H31B—C31—H31C	109.5
C1—C6—H6	119.1	N27—C32—C33	113.02 (18)
C5—C6—H6	119.1	N27—C32—H32A	109.0
C8—C7—C2	126.28 (18)	C33—C32—H32A	109.0
C8—C7—H7	116.9	N27—C32—H32B	109.0
C2—C7—H7	116.9	C33—C32—H32B	109.0
C7—C8—C9	125.12 (18)	H32A—C32—H32B	107.8
C7—C8—H8	117.4	C34—C33—C32	113.10 (19)
C9—C8—H8	117.4	C34—C33—H33A	109.0
C10—C9—C14	116.25 (17)	C32—C33—H33A	109.0
C10—C9—C8	122.97 (17)	C34—C33—H33B	109.0
C14—C9—C8	120.78 (17)	C32—C33—H33B	109.0
C11—C10—C9	122.73 (18)	H33A—C33—H33B	107.8
C11—C10—H10	118.6	C35—C34—C33	111.6 (2)
C9—C10—H10	118.6	C35—C34—H34A	109.3
C10—C11—C12	120.92 (18)	C33—C34—H34A	109.3
C10—C11—H11	119.5	C35—C34—H34B	109.3
C12—C11—H11	119.5	C33—C34—H34B	109.3
N27—C12—C13	122.13 (17)	H34A—C34—H34B	108.0
N27—C12—C11	121.25 (18)	C34—C35—H35A	109.5
C13—C12—C11	116.61 (17)	C34—C35—H35B	109.5
C14—C13—C12	121.41 (17)	H35A—C35—H35B	109.5
C14—C13—H13	119.3	C34—C35—H35C	109.5
C12—C13—H13	119.3	H35A—C35—H35C	109.5
C13—C14—C9	121.96 (18)	H35B—C35—H35C	109.5
C13—C14—H14	119.0	C20—N36—C37	120.84 (17)
C9—C14—H14	119.0	C20—N36—C41	119.81 (16)
C16—C15—C5	125.41 (18)	C37—N36—C41	119.23 (16)
C16—C15—H15	117.3	N36—C37—C38	115.73 (17)
C5—C15—H15	117.3	N36—C37—H37A	108.3
C15—C16—C17	126.66 (18)	C38—C37—H37A	108.3
C15—C16—H16	116.7	N36—C37—H37B	108.3
C17—C16—H16	116.7	C38—C37—H37B	108.3
C22—C17—C18	116.17 (17)	H37A—C37—H37B	107.4
C22—C17—C16	123.50 (17)	C37—C38—C39	111.94 (18)
C18—C17—C16	120.32 (17)	C37—C38—H38A	109.2
C19—C18—C17	122.59 (18)	C39—C38—H38A	109.2
C19—C18—H18	118.7	C37—C38—H38B	109.2
C17—C18—H18	118.7	C39—C38—H38B	109.2
C18—C19—C20	120.91 (17)	H38A—C38—H38B	107.9
C18—C19—H19	119.5	C40—C39—C38	113.42 (19)
C20—C19—H19	119.5	C40—C39—H39A	108.9
N36—C20—C19	122.29 (17)	C38—C39—H39A	108.9
N36—C20—C21	121.04 (18)	C40—C39—H39B	108.9
C19—C20—C21	116.67 (17)	C38—C39—H39B	108.9
C22—C21—C20	121.42 (18)	H39A—C39—H39B	107.7
C22—C21—H21	119.3	C39—C40—H40A	109.5
C20—C21—H21	119.3	C39—C40—H40B	109.5
C21—C22—C17	122.22 (17)	H40A—C40—H40B	109.5
C21—C22—H22	118.9	C39—C40—H40C	109.5
C17—C22—H22	118.9	H40A—C40—H40C	109.5
C1—O23—C24	117.31 (15)	H40B—C40—H40C	109.5
O23—C24—H24A	109.5	N36—C41—C42	115.00 (17)
O23—C24—H24B	109.5	N36—C41—H41A	108.5
H24A—C24—H24B	109.5	C42—C41—H41A	108.5
O23—C24—H24C	109.5	N36—C41—H41B	108.5
H24A—C24—H24C	109.5	C42—C41—H41B	108.5
H24B—C24—H24C	109.5	H41A—C41—H41B	107.5
C4—O25—C26	116.76 (15)	C43—C42—C41	111.94 (17)
O25—C26—H26A	109.5	C43—C42—H42A	109.2
O25—C26—H26B	109.5	C41—C42—H42A	109.2
H26A—C26—H26B	109.5	C43—C42—H42B	109.2
O25—C26—H26C	109.5	C41—C42—H42B	109.2
H26A—C26—H26C	109.5	H42A—C42—H42B	107.9
H26B—C26—H26C	109.5	C42—C43—C44	113.68 (19)
C12—N27—C32	119.36 (17)	C42—C43—H43A	108.8
C12—N27—C28	120.79 (17)	C44—C43—H43A	108.8
C32—N27—C28	118.03 (16)	C42—C43—H43B	108.8
N27—C28—C29	115.31 (17)	C44—C43—H43B	108.8
N27—C28—H28A	108.4	H43A—C43—H43B	107.7
C29—C28—H28A	108.4	C43—C44—H44A	109.5
N27—C28—H28B	108.4	C43—C44—H44B	109.5
C29—C28—H28B	108.4	H44A—C44—H44B	109.5
H28A—C28—H28B	107.5	C43—C44—H44C	109.5
C30—C29—C28	113.11 (17)	H44A—C44—H44C	109.5
C30—C29—H29A	109.0	H44B—C44—H44C	109.5
			
O23—C1—C2—C3	−179.33 (16)	C16—C17—C18—C19	179.89 (19)
C6—C1—C2—C3	−0.7 (3)	C17—C18—C19—C20	0.3 (3)
O23—C1—C2—C7	2.7 (3)	C18—C19—C20—N36	178.10 (19)
C6—C1—C2—C7	−178.67 (17)	C18—C19—C20—C21	−1.5 (3)
C1—C2—C3—C4	1.0 (3)	N36—C20—C21—C22	−177.83 (19)
C7—C2—C3—C4	178.82 (18)	C19—C20—C21—C22	1.7 (3)
C2—C3—C4—O25	−179.89 (17)	C20—C21—C22—C17	−0.9 (3)
C2—C3—C4—C5	−1.3 (3)	C18—C17—C22—C21	−0.3 (3)
O25—C4—C5—C6	−179.96 (16)	C16—C17—C22—C21	−179.60 (19)
C3—C4—C5—C6	1.4 (3)	C6—C1—O23—C24	8.2 (3)
O25—C4—C5—C15	−1.3 (3)	C2—C1—O23—C24	−173.22 (17)
C3—C4—C5—C15	−179.98 (17)	C3—C4—O25—C26	−10.5 (3)
O23—C1—C6—C5	179.36 (18)	C5—C4—O25—C26	170.81 (17)
C2—C1—C6—C5	0.9 (3)	C13—C12—N27—C32	−9.5 (3)
C4—C5—C6—C1	−1.2 (3)	C11—C12—N27—C32	171.42 (19)
C15—C5—C6—C1	−179.79 (18)	C13—C12—N27—C28	−173.84 (18)
C3—C2—C7—C8	21.3 (3)	C11—C12—N27—C28	7.1 (3)
C1—C2—C7—C8	−160.87 (19)	C12—N27—C28—C29	75.8 (2)
C2—C7—C8—C9	−176.34 (18)	C32—N27—C28—C29	−88.7 (2)
C7—C8—C9—C10	17.1 (3)	N27—C28—C29—C30	−166.59 (17)
C7—C8—C9—C14	−163.12 (19)	C28—C29—C30—C31	72.4 (2)
C14—C9—C10—C11	2.3 (3)	C12—N27—C32—C33	−72.8 (2)
C8—C9—C10—C11	−177.87 (19)	C28—N27—C32—C33	91.9 (2)
C9—C10—C11—C12	0.5 (3)	N27—C32—C33—C34	159.90 (19)
C10—C11—C12—N27	175.96 (19)	C32—C33—C34—C35	−177.72 (19)
C10—C11—C12—C13	−3.1 (3)	C19—C20—N36—C37	−8.3 (3)
N27—C12—C13—C14	−176.10 (19)	C21—C20—N36—C37	171.27 (18)
C11—C12—C13—C14	3.0 (3)	C19—C20—N36—C41	175.79 (18)
C12—C13—C14—C9	−0.2 (3)	C21—C20—N36—C41	−4.7 (3)
C10—C9—C14—C13	−2.5 (3)	C20—N36—C37—C38	89.4 (2)
C8—C9—C14—C13	177.72 (18)	C41—N36—C37—C38	−94.6 (2)
C6—C5—C15—C16	−14.9 (3)	N36—C37—C38—C39	−174.26 (18)
C4—C5—C15—C16	166.46 (19)	C37—C38—C39—C40	173.5 (2)
C5—C15—C16—C17	176.70 (18)	C20—N36—C41—C42	−73.6 (2)
C15—C16—C17—C22	−2.8 (3)	C37—N36—C41—C42	110.4 (2)
C15—C16—C17—C18	177.90 (19)	N36—C41—C42—C43	159.66 (17)
C22—C17—C18—C19	0.5 (3)	C41—C42—C43—C44	177.43 (19)

### N,N-Dibutyl-4-[(E)-2-(4-{(E)-2-[4-(dibutylamino)phenyl]ethenyl}-2,5-dimethoxyphenyl)ethenyl]aniline Hydrogen-bond geometry (Å, º)

*Cg*3 is the centroid of the C17–C22 ring.

**Table:**

*D*—H···*A*	*D*—H	H···*A*	*D*···*A*	*D*—H···*A*
C30—H30*B*···Cg3^i^	0.99	2.78	3.719 (2)	158

Symmetry code: (i) *x*−1, *y*, *z*−1.
